# The cranberry flavonoids PAC DP-9 and quercetin aglycone induce cytotoxicity and cell cycle arrest and increase cisplatin sensitivity in ovarian cancer cells

**DOI:** 10.3892/ijo.2015.2931

**Published:** 2015-03-17

**Authors:** YIFEI WANG, ALEX HAN, EVA CHEN, RAKESH K. SINGH, CLINTON O. CHICHESTER, RICHARD G. MOORE, AJAY P. SINGH, NICHOLI VORSA

**Affiliations:** 1 Department of Plant Biology and Pathology, Rutgers University, New Brunswick, NJ 08901, USA; 2 Molecular Therapeutics Laboratory, Program in Women’s Oncology, Women and Infants’ Hospital of Rhode Island, Alpert Medical School, Brown University, Providence, RI 02905, USA; 3 College of Pharmacy, University of Rhode Island, Kingston, RI 02881, USA; 4 Philip E. Marucci Center for Blueberry and Cranberry Research and Extension, Rutgers University, Chatsworth, NJ 08019, USA

**Keywords:** cranberry, flavonoids, ovarian cancer, apoptosis, cell cycle

## Abstract

Cranberry flavonoids (flavonols and flavan-3-ols), in addition to their antioxidant properties, have been shown to possess potential *in vitro* activity against several cancers. However, the difficulty of isolating cranberry compounds has largely limited anticancer research to crude fractions without well-defined compound composition. In this study, individual cranberry flavonoids were isolated to the highest purity achieved so far using gravity and high performance column chromatography and LC-MS characterization. MTS assay indicated differential cell viability reduction of SKOV-3 and OVCAR-8 ovarian cancer cells treated with individual cranberry flavonoids. Treatment with quercetin aglycone and PAC DP-9, which exhibited the strongest activity, induced apoptosis, led to caspase-3 activation and PARP deactivation, and increased sensitivity to cisplatin. Furthermore, immunofluorescence microscopy and western blot study revealed reduced expression and activation of epidermal growth factor receptor (EGFR) in PAC DP-9 treated SKOV-3 cells. In addition, quercetin aglycone and PAC DP-9 deactivated MAPK-ERK pathway, induced downregulation of cyclin D1, DNA-PK, phosphohistone H3 and upregulation of p21, and arrested cell cycle progression. Overall, this study demonstrates promising *in vitro* cytotoxic and anti-proliferative properties of two newly characterized cranberry flavonoids, quercetin aglycone and PAC DP-9, against ovarian cancer cells.

## Introduction

Epithelial ovarian cancer (EOC) is an asymptotic lethal disease that is frequently diagnosed at late stages in women (1). Initial responsiveness to chemotherapy and surgery is often thwarted by inherent or acquired resistance, resulting in early recurrence and premature death (2). Approximately 22,000 women will be diagnosed with EOC this year in the US alone, and more than 14,000 will succumb to their disease (3). Because first-line platinum-taxane and second-line dox-topotecan therapies often fail, third-line chemotherapy options are urgently needed.

American cranberry (*Vaccinium macrocarpon*) has received attention in our laboratories and elsewhere because of the potential for cranberry A-type proanthocyanidins (PACs) and flavonols to treat upper urinary tract infections and cancer (4–6). However, the isolation of pure PACs and flavonol constituents has remained an unmet challenge that frequently impedes broad-spectrum screening for biological activity. Semi-pure cranberry PACs have consistently exhibited potent anti-proliferative activity against various cancer cells *in vitro* (6–8). We had previously developed an iterative but efficient HPLC and mass spectrometry-based approach to generate high-purity polymeric PAC fractions from cranberries (9). Purified PACs have exhibited cytotoxic effects against a panel of gynecologic cancer and neuroblastoma cells in our laboratories (9–11). PACs exerted these cytotoxic effects via cell cycle arrest, production of lethal levels of intracellular reactive oxygen species (ROS), and induction of pro-apoptotic signal transductions at low microgram concentrations (10,11). Further optimization of the purification and a detailed investigation of the mechanism of anti-proliferative action have been pursued in our laboratories since purified PACs became accessible.

In this study, we further elaborate analytical methodology to isolate and purify individual flavonols and PACs of cranberry for broad-spectrum biological activity screening studies. We also describe the two most active leads, PAC DP-9 and quercetin aglycone, in SKOV-3 and OVCAR-8 ovarian cancer cells, and we characterize their anti-proliferative efficacy and mechanism of cell cycle arrest, induction of apoptotic activities, and inhibition of oncogenes and DNA repair machinery. The multifaceted anti-proliferative properties exerted by these two cranberry flavonoids highlight their potential for treatment of ovarian cancer.

## Materials and methods

### Plant material

Cranberry fruits of cultivar ‘Stevens’ were harvested from the Philip E. Marucci Center for Blueberry and Cranberry Research and Extension and kept frozen at −20°C before use.

### Reagents and LC-MS instrumentation

All solvents were purchased from EMD Millipore (Billercia, MA, USA). Sephadex^®^ LH-20 was obtained from GE Healthcare Bio-Science (Piscataway, NJ, USA), and BakerBound^®^ Diol was obtained from Avantor Performance Materials (Center Valley, PA, USA). LC-MS spectra were obtained with a Dionex UltiMate^®^ 3000 LC system (Thermal Scientific, Sunnyvale, CA, USA) including the UltiMate 3000 RS Pump, UltiMate 3000 RS Autosampler, UltiMate 3000 RS Column Compartment and UltiMate 3000 RS Diode Array Detector coupled with Applied Biosystems API 3000^TM^ triple quad LC-MS/MS mass spectrometer (AB SCIEX, Framingham, MA, USA). Previously described HPLC methods for flavonol and PAC identification (12,13) were modified slightly for LC-MS analysis. Structure and purity of flavonols and PACs were determined by HPLC-PDA/Fluorescence and/or LC-MS.

### Extraction and isolation of individual cranberry flavonols and PACs

Crude flavonoids were extracted and further separated in a Sephadex LH-20 column as previously described (14). Individual cranberry flavonols were isolated using a semi-preparative HPLC system as described previously (14). Individual PACs were isolated with a regular Diol gravity column chromatography as previously reported (9). Eight flavonols were isolated and characterized as myricetin-3-galactoside, quercetin-3-galactoside, quercetin-3-glucoside, quercetin-3-xylopyranoside, quercetin-3-arabinopyranosdie, quercetin-3-arabinofuranoside, quercetin-3-rhamnopyranoside and quercetin aglycone. Eleven cranberry A-type PACs from dimer to polymer 12 (named as PAC DP-2 to PAC DP-12) were isolated and characterized. Purity of all isolated cranberry flavonoids was > 95% (w/w) based on HPLC and LC-MS analysis.

### Cell lines and cell culture

SKOV-3 and OVCAR-8 cells (ovarian epithelial adenocarcinoma) were purchased from ATCC (Manassas, VA, USA). Cells were cultured with Dulbecco’s modified Eagle’s medium (DMEM, Life Technologies, Carlsbad, CA, USA) supplemented with 10% fetal bovine serum (Life Technologies), 100 μg/ml streptomycin and 100 μg/ml penicillin (Life Technologies) in an incubator at 37°C, 5% CO_2_ and 95% humidity. For all assays, cells were allowed to attach for 24 h prior to treatment.

### Cell viability assay

Cells (5,000/well) were seeded in 96-well flat bottom plates (USA Scientific, Orlando, FL, USA) and treated with various concentrations of flavonoids for 72 h. Cell viability was determined by CellTiter 96^®^ Aqueous One Solution assay (Promega, Madison, WI, USA) following the manufacturer’s protocol. Experiments were performed in triplicate; data are expressed as mean of triplicate measurements (mean ± SD) in percentage of untreated cells (100%). SPSS Statistics 19 (IBM Corp., Armonk, NY, USA) was used to perform ANOVA with linear regression between cell viability and compound concentration, calculate IC_50_ value of each cranberry flavonoid, and conduct Student’s t-tests and calculate p-values based on mean cell viability for each treatment.

### DNA fragmentation analysis

DNA fragmentation as a hallmark of apoptosis was studied using the Roche *In Situ* Cell Death Detection kit (Branford, CT, USA) (TUNEL assay). SKOV-3 and OVCAR-8 cells (2×10^4^/well) were seeded in Lab-Tek 8-well chamber glass slides (Nalge Nunc., Naperville, IL, USA) and treated with 50 μg/ml PAC DP-9, 25 μg/ml quercetin aglycone, or DMSO vehicle control for 12 h. Cells were fixed with 10% neutral buffered formalin, and stained according to the manufacturer’s protocol. Slides were then cover-slipped with Vectashield mounting medium with DAPI (Vector Laboratories, Burlingame, CA, USA). Images were acquired on a Nikon E800 upright microscope (Nikon Instruments, Inc., Melville, NY, USA) using SPOT Advanced Software (SPOT Imaging Solutions, Sterling Heights, MA, USA).

### Western blot analysis

Cells (3×10^6^/dish) were seeded into 100-mm^2^ tissue culture dishes and treated with individual cranberry flavonoids. Cells were then lysed with cell lysis buffer (Cell Signaling Technology, Inc., Danvers, MA, USA) supplemented with 5 μl/ml phenylmethylsulfonyl fluoride (Sigma-Aldrich, St. Louis, MO, USA). Protein concentrations of cell lysates were determined with Pierce BCA protein assay kit (Pierce Technology, Rockford, IL, USA). Gel electrophoresis was performed in NuPAGE Gel system (Life Technologies) according to the manufacturer’s instructions. Separated proteins were transferred onto a nitrocellulose membrane, which was then blocked with 5% non-fat milk in PBS-Tween buffer and probed against various primary antibodies (cleaved caspase-3 no. 9664, cleaved PARP no. 5625, EGFR no. 4267, phospho-EGFR no. 3777, phospho-c-Raf no. 9427, phospho-ERK1/2 no. 4370, phospho-p53 no. 9286, p18 INK4C no. 2896, p21 Waf1/Cip1 no. 2947, p27 Kip1 no. 3686, CDK2 no. 2546, cyclin D1 no. 2926, cyclin D3 no. 2936, β-actin no. 8457, β-tubulin no. 2128; Cell Signaling Technology). Protein bands were visualized using horseradish peroxidase conjugated anti-rabbit or anti-mouse secondary antibody (Cell Signaling Technology) and Pierce ECL Western Blotting Substrate, and documented by Bio-Rad Gel Doc system (Bio-Rad, Hercules, CA, USA).

### Immunofluorescence microscopy analysis

SKOV-3 and OVCAR-8 cells (2×10^4^/well) were seeded in Lab-Tek 8-well chamber slides and treated with individual cranberry flavonoids overnight for 12 h. Cells were fixed with 10% neutral buffered formalin, washed 3 times with PBS-Tween 0.1% and blocked with 5% horse serum (Vector Laboratories) in PBS-Tween for 30 min. Blocked cells were incubated with various primary antibodies (EGFR no. 4267, p21 Waf1/Cip1 no. 2947, phospho-ERK1/2no.4370,DNA-PKno.4602, Cell Signaling Technology; and MEK no. sc-166197, phospho-histone H3 no. sc-12927, cyclin D1 no. sc-246, Santa Cruz Biotechnology, Dallas, TX, USA) at 4°C overnight. Probed proteins were visualized with DyLight 488/594 (Thermo Scientific, Waltham, MA, USA) or Alexa Fluor^®^ 594 (Cell Signaling Technology) secondary antibodies, and nuclei were stained with Vectorshield^®^ mounting medium with DAPI (Vector Laboratories). Images were acquired on Olympus FSX100^®^ (Olympus America Inc., Center Valley, PA, USA; for EGFR) or Nikon E800 upright (for other proteins) microscope system.

### Co-immunoprecipitation analysis

SKOV-3 and OVCAR-8 cells were cultured in 100 mm^2^ tissue culture dishes to 80% confluency. Cells were lysed and protein concentration was quantified as described earlier. Lysate containing 500 μg protein was incubated with target antibody or control IgG antibody (Cell Signaling Technology) for 4 h with rotation at 4°C. Protein G sepharose (75 μl) (50% slurry, GE Healthcare Life Sciences) was added to the lysate and incubated at 4°C overnight. After incubation, beads were washed with 500 μl lysis buffer 3 times and re-suspended in 40 μl Laemmli buffer (2X, Bio-Rad), vortexed, and heated to 95°C for 5 min. Suspension was centrifuged to collect supernatant for western blot analysis.

### Cell cycle analysis

SKOV-3 and OVCAR-8 cells (3×10^5^/well) were seeded in 6-well plates and treated with a series of concentrations of individual cranberry flavonoids (0–100 μg/ml, 24–48 h). After trypsinization, cells were collected and fixed in ice-cold 70% ethanol and stained with solution containing propidium iodide (Sigma-Aldrich; 0.1 mg/ml), sodium citrate (Sigma-Aldrich; 2 mg/ml) and Triton X-100 (Sigma-Aldrich; 1 μl/ml). Cell counting data were acquired in an Accuri^®^ C6 Flow Cytometer (BD Biosciences, San Jose, CA, USA) and analyzed with ModFit LT software (Verity Software House, Inc., Topsham, ME, USA).

## Results

### Individual cranberry flavonoids display differential cytotoxicity against ovarian cancer cell lines

Individual cranberry flavonols and PACs exhibited different levels of cytotoxicity against SKOV-3 and OVCAR-8 cell-lines (Fig. 2). Quercetin-3-xylopyranoside did not show cytotoxicity against SKOV-3 and OVCAR-8 cells, while myricetin-3-galactoside and quercetin-3-arabinopyranoside were cytotoxic to OVCAR-8 cells (IC_50_, 130 and 212 μg/ml) but non-toxic to SKOV-3 cells at treatment concentrations. Compared to quercetin glycosides, quercetin aglycone exhibited higher cytotoxicity against the two cancer cell lines (IC_50_, 83 and 61 μg/ml for SKOV-3 and OVCAR-8 cells, respectively).

### Cranberry PAC DP-3, DP-7, and DP-10 exhibited less cytotoxicity against both SKOV-3 and OVCAR-8 cells than other PACs

**Figure 1:**
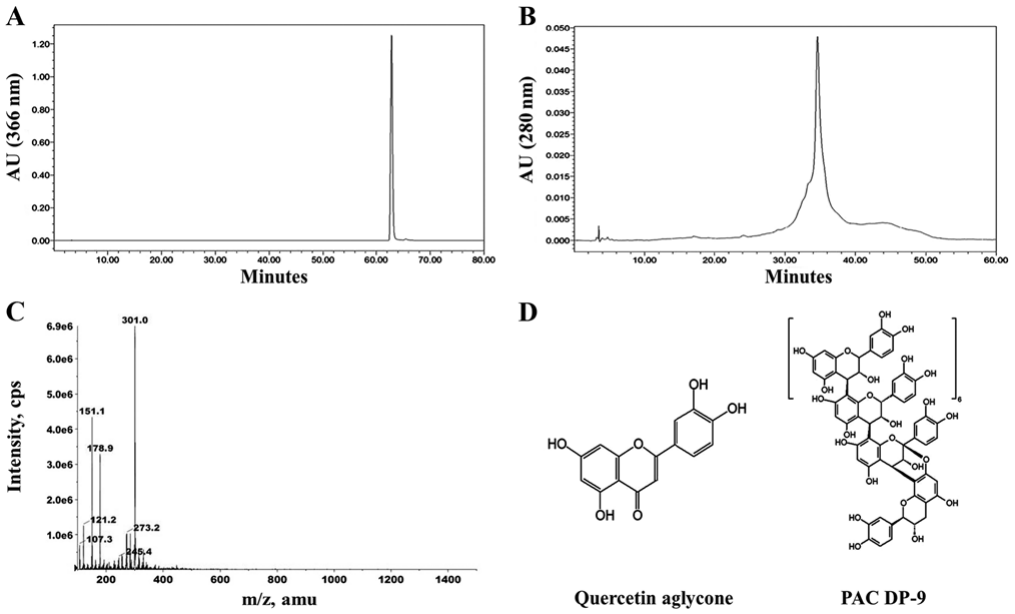
Structures of cranberry flavonoids quercetin aglycone and PAC DP-9. (A) HPLC chromatograph of quercetin aglycone; (B) HPLC chromatograph of PAC DP-9; (C) MS spectrum of quercetin aglycone (m/z=301); (D) Structures of quercetin algycone and PAC DP-9.

**Figure 2:**
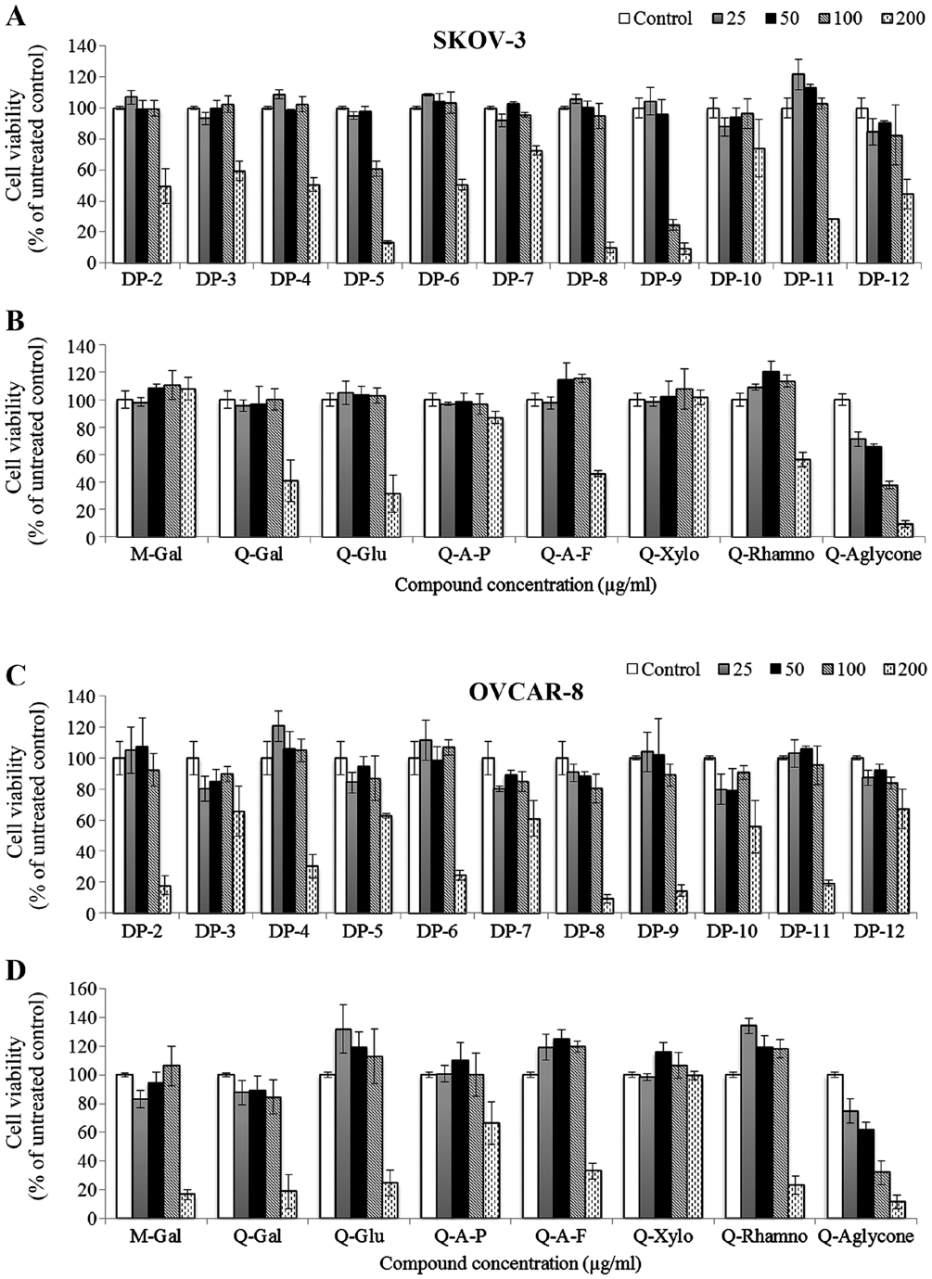
Cytotoxicity of individual cranberry flavonols and PACs in SKOV-3 and OVCAR-8 ovarian cancer cells. Cytotoxicity was determined by MTS cell viability assay; cells were treated with DMSO vehicle or different concentrations of cranberry flavonoids (25–200 μg/ml) for 72 h. Experiments were performed in triplicate; data are expressed as mean ± SD in percent of cell viability of untreated cells (100%). M-Gal, myricetin-3-galactoside; Q-Gal, quercetin-3-galactoside; Q-Glu, quercetin-3-glucoside; Q-A-P, quercetin-3-arabinopyranoside; Q-A-F, quercetin-3-arabinofuranoside; Q-Xylo, quercetin-3-xylopyranoside; Q-Rhamno, quercetin-3-rhamnopyranoside;Q-Aglycone, quercetin aglycone.

PAC DP-5 and DP-12 were more effective against SKOV-3 cells (IC_50_, 126 μg/ml and 162 μg/ml for DP-5 and DP-12, respectively) than OVCAR-8 cells. Compared to other cytotoxic PAC molecules, PAC DP-9 exhibited the highest activity against SKOV-3 cells (IC_50_, 82 μg/ml) and relatively high cytotoxicity against OVCAR-8 cells (IC_50_, 138 μg/ml). Based on these results, quercetin aglycone and PAC DP-9 (Fig. 1) were selected as lead candidates for our studies in SKOV-3 and OVCAR-8 cells. Cytotoxicity values for individual flavonoids and statistical analysis including p-values and correlation coefficients are provided in Table I.

### Quercetin aglycone and PAC DP-9 induced apoptosis and increased cisplatin sensitivity in SKOV-3 and OVCAR-8 cells

**Figure 3:**
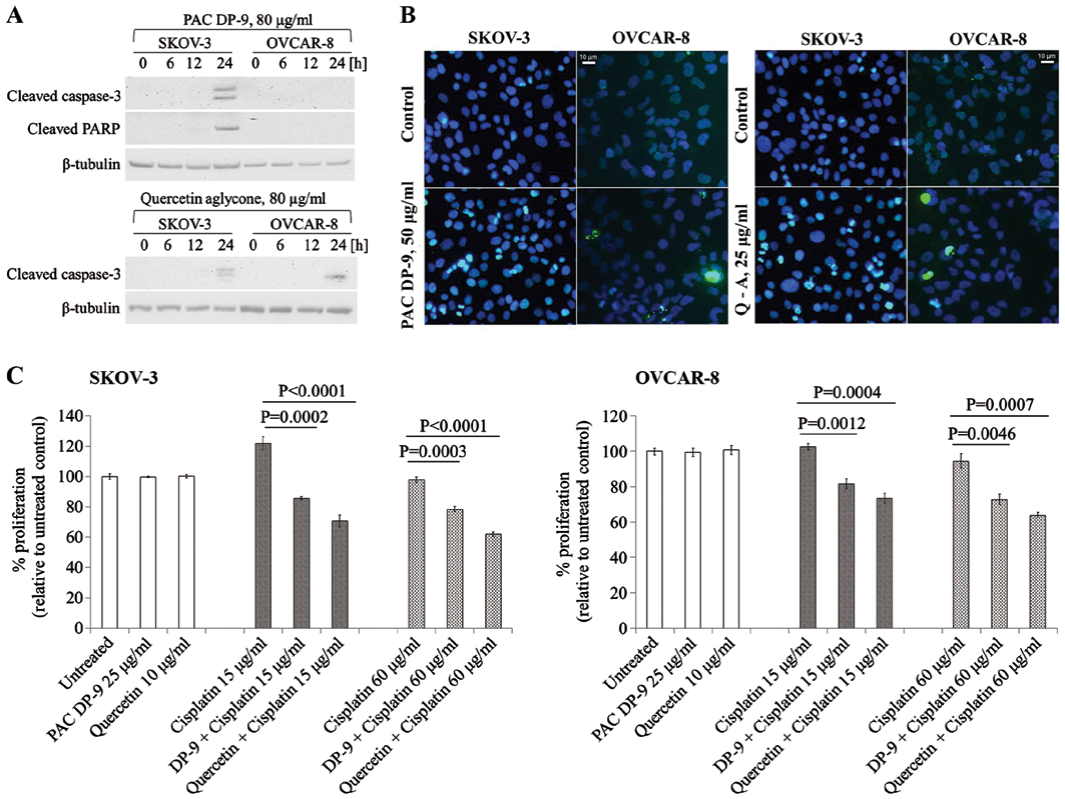
Induction of apoptosis (A and B) and sensitization to cisplatin (C) by quercetin aglycone and PAC DP-9 in SKOV-3 and OVCAR-8 cells. (A) Expression of cleaved caspase-3 and cleaved PARP after treatment with PAC DP-9 and quercetin aglycone. Ovarian cancer cells were treated with PAC DP-9 or quercetin aglycone (80 μg/ml) for 0–24 h. Actin or tubulin were probed as internal loading controls; (B) TUNEL assay in PAC DP-9 and quercetin aglycone (Q-A)-treated SKOV-3 and OVCAR-8 cells. Cells were treated with PAC DP-9 (50 μg/ml), quercetin aglycone (25 μg/ml) or DMSO vehicle for 12 h. DNA strand breaks due to apoptosis were detected and labelled by fluorescein-labelled nucleotides (green). (C) Cell viability of cranberry flavonoid and cisplatin-treated SKOV-3 and OVCAR-8 cells. Cells were pre-treated with either DMSO vehicle, PAC DP-9 (25 μg/ml), or quercetin aglycone (10 μg/ml) for 6 h in complete DMEM media, followed by cisplatin treatment alone or cisplatin + PAC DP-9/quercetin aglycone. Cells were incubated overnight for 12 h and analyzed via MTS for viability. Experiments were performed in triplicate; data are expressed as mean ± SD in percent of cell viability of untreated cells (100%).

To examine whether apoptosis was induced in ovarian cancer cells upon treatment with cranberry flavonoids, western blot and DNA fragmentation analysis (TUNEL assay) were carried. As shown in Fig. 3A, quercetin aglycone induced caspase-3 activation in both SKOV-3 and OVCAR-8 cells, and PAC DP-9 led to caspase-3 and cleaved-PARP expression specifically in SKOV-3 cells.

TUNEL assay was performed to detect apoptosis-induced DNA fragmentation. Quercetin aglycone and PAC DP-9 treated cells showed apoptosis-induced DNA fragmentation, which was detected in both SKOV-3 and OVCAR-8 cells after 12 h of treatment (Fig. 3B). Both western blot and DNA fragmentation analysis confirmed apoptosis induction in ovarian cancer cells exposed to these two cranberry flavonoids.

To investigate whether quercetin aglycone and PAC DP-9 could sensitize ovarian cancer cells to cisplatin, cells were pretreated with subtoxic concentrations of quercetin aglycone or PAC DP-9 for 6 h, exposed to cisplatin overnight for 12 h, and cell viability was analyzed. As shown in Fig. 3C, while subtoxic concentrations of quercetin aglycone or PAC DP-9 alone did not reduce SKOV-3 and OVCAR-8 cell viability, their pretreatment significantly reduced viability of cisplatin-treated SKOV-3 and OVCAR-8 cells, with quercetin aglycone acting more strongly than PAC DP-9. Thus, quercetin aglycone and PAC DP-9 at low concentrations sensitize the response of cisplatin-resistant ovarian cancer cells to cisplatin, resulting in enhanced cytotoxicity.

### PAC DP-9 downregulated expression and activation of epidermal growth factor receptor (EGFR) in SKOV-3 cells and induced EGFR nuclear translocation

**Figure 4:**
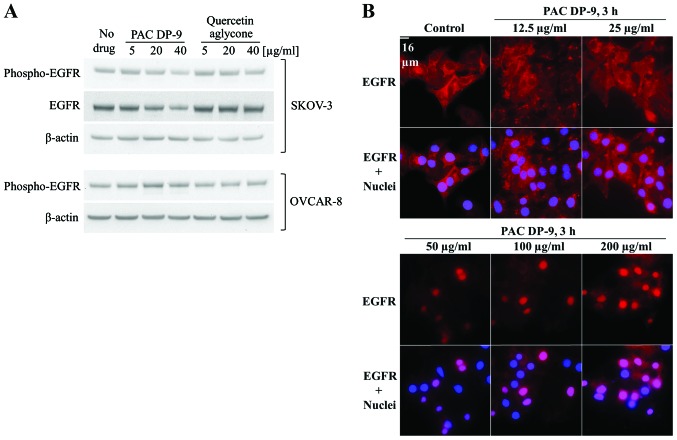
Effects of quercetin aglycone and PAC DP-9 on expression, activation and localization of EGFR in SKOV-3 and OVCAR-8 cells. (A) Expression of total and activated EGFR on cranberry flavonoid treated SKOV-3 and OVCAR-8 cells. Cells were serum-deprived for 4 h and then treated with 5, 20 or 40 μg/ml quercetin aglycone or PAC DP-9 in serum-free DMEM medium for 12 h. Actin was probed as internal loading control. (B) Effects of PAC DP-9 on EGFR expression and localization in SKOV-3 cells. Cells were treated with different concentrations (12.5–200 μg/ml) of PAC DP-9 for 3 h before fluorescent microscopic analysis. EGFR was visualized by Alexa Fluor 594 secondary antibody (red), and nuclei were stained with DAPI.

Elevated EGFR expression results in poor prognosis in lung, breast and ovarian cancer (15). EGFR expression in quercetin aglycone or PAC DP-9 treated ovarian cancer cells was analyzed by immunoblotting and immunofluorescence microscopy. To eliminate the effect of serum growth factors on cellular EGFR regulation, ovarian cancer cells were serum-deprived for 4 h prior to treatment, and low concentrations (5–40 μg/ml) of cranberry flavonoids were applied with serum-free medium to avoid cell toxicity. After 12 h of treatment, quercetin aglycone did not affect phosphorylated-EGFR levels in either SKOV-3 or OVCAR-8 cells, but PAC DP-9 induced a dose-dependent downregulation of both phosphorylated-EGFR and total EGFR in SKOV-3 cells (Fig. 4A). As shown in Fig. 4B, EGFR was expressed predominantly at the cell membrane in the untreated controls, and exhibited nuclear translocation within 3 h of treatment with PAC DP-9 in a dose-dependent manner such that lower concentrations (12.5–25 μg/ml) induced EGFR peri-nuclear localization in SKOV-3 cells, and higher concentrations (50–200 μg/ml) of PAC DP-9 mediated EGFR nuclear translocation.

### Quercetin aglycone and PAC DP-9 downregulated pro-survival MAP kinase proteins in ovarian cancer cells

**Figure 5:**
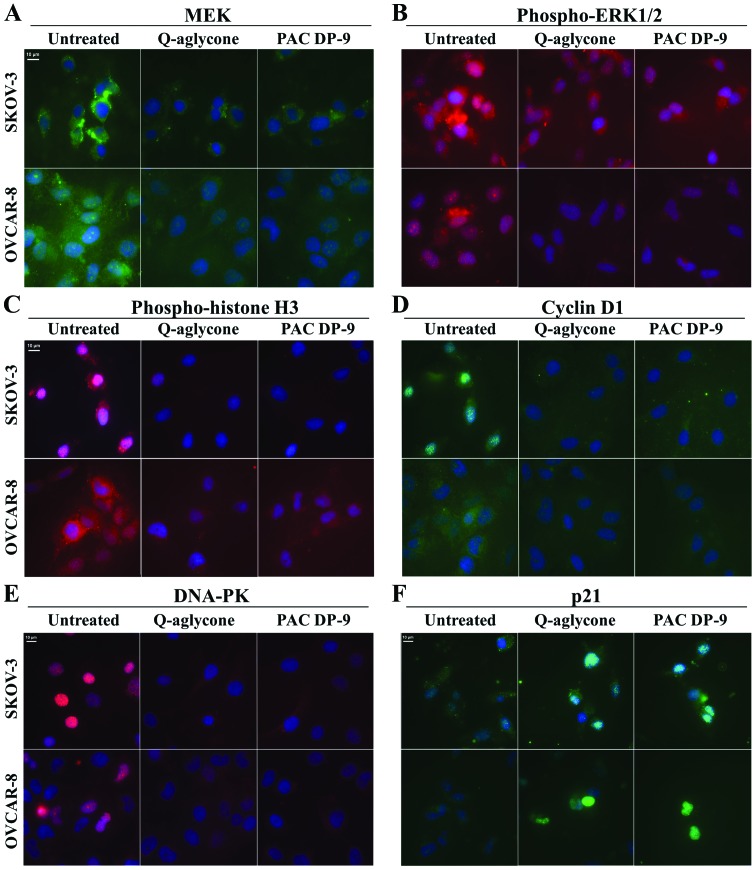
Effect of quercetin aglycone and PAC DP-9 (12-h treatment) on cellular expression of MEK (A), phospho-ERK1/2 (B), phospho-histone H3 (C), cyclin D1 (D), DNA-PK (E) and p21 (F) in SKOV-3 and OVCAR-8 cells. Cells were treated with either DMSO vehicle, PAC DP-9 (80 μg/ml), or quercetin aglycone (80 μg/ml) for 12 h and stained for target proteins. MEK, cyclin D1 and p21 were probed by DyLight 488 secondary antibody (green); phospho-ERK1/2, phospho-histone H3 and DNA-PK were probed by DyLight 594 secondary antibody (red).

**Figure 6:**
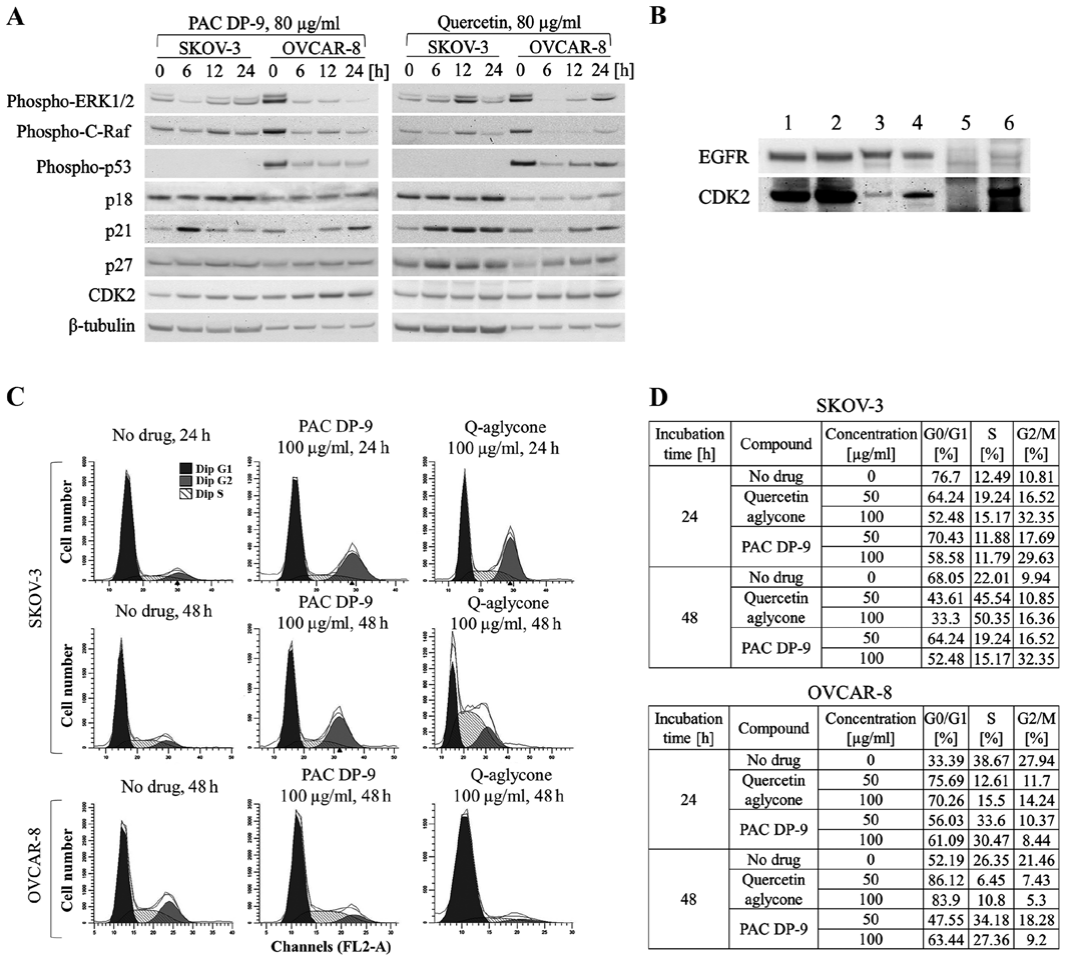
Effect of PAC DP-9 and quercetin aglycone on cell cycle regulation in SKOV-3 and OVCAR-8 cells. (A) Western blot analysis of ERK-MAPK signal components and cell cycle regulatory factors in SKOV-3 and OVCAR-8 cells after treatment with quercetin aglycone or PAC DP-9 (80 μg/ml) for 0–24 h. Tubulin was probed as an internal loading control; (B) EGFR co-immunoprecipitation on SKOV-3 and OVCAR-8 cell lysates. 1, SKOV-3 untreated input control; 2, OVCAR-8 untreated input control; 3, SKOV-3 EGFR-co-immunoprecipitation; 4, OVCAR-8 EGFR-co-immunoprecipitation; 5, SKOV-3 IgG-co-immunoprecipitation control; 6, OVCAR-8 IgG-co-immunoprecipitation control. (C and D) Determination of cell cycle progression by FACS analysis in SKOV-3 and OVCAR-8 cells treated with 50 or 100 μg/ml quercetin aglycone or PAC DP-9 for 24–48 h. Data are presented as relative fluorescence intensity of G0/G1, S and G2/M phase subpopulations in 2-D charts (C) or tables (D).

We examined changes in MAP kinase signaling regulation exerted by quercetin aglycone and PAC DP-9 in ovarian cancer cells. Expression of MEK (Fig. 5A) and phospho-ERK1/2 (Fig. 5B) was downregulated after 12 h of treatment with quercetin aglycone or PAC DP-9, indicating inhibition of pro-survival MAPK-ERK signal transduction by cranberry flavonoids. Similarly, treatment with quercetin aglycone and PAC-9 led to rapid, significant downregulation of phospho-p42/22 MAPK and phospho-c-Raf in OVCAR-8 cells within 6 h (Fig. 6A), demonstrating their inhibitory effect on the activated pro-survival MAPK pathway.

### Quercetin aglycone and PAC DP-9 affected cell cycle progression of ovarian cancer cells

Immunofluorescence microscopy analysis of quercetin aglycone and PAC DP-9 treatment revealed downregulation of cyclin D1 and upregulation of p21 in SKOV-3 and OVCAR-8 cells (Fig. 5D and F). Phospho-histone H3 (Fig. 5C) and DNA-dependent protein kinase (DNA-PK, Fig. 5E), proteins often overexpressed in ovarian cancer cells, were also downregulated in SKOV-3 and OVCAR-8 cells upon treatment with cranberry flavonoids. Based on immunoblot study, phospho-p53 and p21 expression were first downregulated after 6 h of treatment with quercetin aglycone in OVCAR-8 cells and then exhibited sustained upregulation (Fig. 6A). Three other cell cycle regulators, p18, p27, and CDK2 were also upregulated within 6–24 h of treatment with quercetin aglycone. Independent of p53 expression, SKOV-3 cells exhibited rapid upregulation of cell cycle inhibitor p21 after 6 h of treatment of quercetin aglycone, together with upregulation of p18, p27, and CDK2 within 24 h of treatment. Similarly, PAC DP-9 induced upregulation of p21 in both SKOV-3 and OVCAR-8 cells within 6–24 h-treatment regardless of p53 expression level. CDK2 expression was also upregulated in SKOV-3 and OVCAR-8 cells after 24 h of PAC DP-9 treatment.

Because immunofluorescence microscopy and western blot analysis showed inhibition of SKOV-3 cellular EGFR expression by PAC DP-9 (Fig. 4), co-immunoprecipitation (Co-IP) was utilized to examine potential protein-protein interactions involved with EGFR and cell cycle regulatory factors, including p18, p21, p27, CDK2, cyclin D1 and cyclin D3. While most of the probed proteins did not show a positive signal, CDK2 signal was detected in both SKOV-3 and OVCAR-8 EGFR Co-IP samples. As shown in Fig. 6B, CDK2 was recovered in both positive controls and two Co-IP samples as well as in the OVCAR-8 negative control, indicating a false-positive signal in the OVCAR-8 Co-IP sample. The absence of CDK2 signal in SKOV-3 negative control confirmed direct interaction between CDK2 and EGFR in SKOV-3 cells.

Both western blot and Co-IP studies indicate a direct effect of cranberry flavonoids on ovarian cancer cell progression. Subpopulations of propidium iodide-stained SKOV-3 and OVCAR-8 ovarian cancer cells treated with cranberry flavonoids were analyzed by flow cytometry. As illustrated in Fig. 6C and D, quercetin aglycone or PAC DP-9 treatment for 24 h in SKOV-3 cells led to G2/M-phase arrest dose-dependently. The G2/M subpopulation increased from 10.81 to 16.52% for SKOV-3 cells treated with 50 μg/ml quercetin aglycone and further increased to 32.35% after treatment with 100 μg/ml quercetin aglycone. Similarly, 50 and 100 μg/ml PAC DP-9 caused an increase in the SKOV-3 cell G2/M subpopulation to 17.69 and 29.63%, respectively. After 48 h of treatment in SKOV-3 cells, PAC DP-9 exhibited similar dose-dependent G2/M-phase arrest, whereas 50 and 100 μg/ml quercetin aglycone led to an increase in S subpopulation from 22.01 to 45.54 and 50.35%, respectively, with no significant change in G2/M subpopulation. This suggests that quercetin aglycone caused S/G2-phase arrest in SKOV-3 cells after 48 h of treatment.

Quercetin aglycone treatment for 24 and 48 h in OVCAR-8 cells caused G1/S-phase arrest. At 50 μg/ml, quercetin aglycone led to retention of 75.69 and 86.12% of cells in G0/G1 phase after 24 and 48 h of treatment, respectively, and the subpopulation of S-phase cells decreased from 38.67 to 12.61% after 24 h and from 26.35 to 6.45% after 48 h (Fig. 6D). PAC DP-9 showed a similar effect in OVCAR-8 cells, thus confirming that G1/S-phase arrest was caused by the two compounds in OVCAR-8 ovarian cancer cells.

## Discussion

Cranberry phenolic extracts have shown *in vitro* anticancer and chemo-preventive properties in different cancer cell lines (5–11). Research on individual phenolic compounds of cranberry has been limited by the difficulty of compound isolation due to complex structural variations, including degree-of-polymerization, linkage type, position of the double linkage between constituent units, and attachment with sugar moieties (9,12). Serial application of Dionex UltiMate^®^ 3000 LC system consisting of UltiMate 3000 RS Pump, UltiMate 3000 RS Autosampler, UltiMate 3000 RS Column Compartment, and UltiMate 3000 RS Diode Array Detector coupled with Applied Biosystems API 3000 triple quad LC-MS/MS mass spectrometer has allowed the reproducible isolation and characterization of individual PACs with up to the 10th degree of polymerization in >95% purity in our laboratories. So far, we have successfully isolated 19 high-purity individual cranberry flavonoids, characterized their structures by LC-APCI-MS, and used them as primary materials to determine bioactivity.

SKOV-3 and OVCAR-8 ovarian cell lines, which possess several key oncogenic hallmarks such as p53 mutation, EGFR overexpression, and cisplatin resistance (16–18), were employed as an *in vitro* cell culture model to determine the mechanism of action of cranberry flavonoids. Individual PACs DP-2 to DP-4 failed to show strong cytotoxicity against SKOV-3 cells, and high molecular weight PAC polymers generally exhibited stronger cytotoxicity similar to what we showed earlier (9). Quercetin aglycone emerged as the most cytotoxic of the tested cranberry flavonols. Shen *et al* showed that quercetin aglycone exhibited significantly higher cytotoxicity against human promyelocytic leukemia cells HL-60 compared to its rutinose and rhamnose glycosides (19). Murota *et al* also reported that quercetin aglycone was much more efficiently absorbed by Caco-2 colorectal adenocarcinoma cells than its glycosides (20). Both quercetin aglycone and PAC DP-9 induced ovarian cancer cell death through apoptotic events, and subtoxic concentrations of the two compounds significantly increased the efficacy of cisplatin against ovarian cancer cells. Because developed resistance to platinum-based drugs is one of the major causes of mortality in ovarian cancer (2), the observation that cranberry flavonoids can sensitize drug-resistant ovarian cancer cells to cisplatin provides an opportunity to improve the efficacy of platinum chemotherapies and reduce side effects associated with cisplatin.

EGFR, a receptor tyrosine kinase, is involved in many signaling pathways that modulate cell survival, proliferation and apoptosis. Aberrant activation of EGFR has been shown to play a critical role in cancer cell survival and development (18,21). B-type PACs isolated from grape seeds have been shown to target and downregulate EGFR expression in human head and neck squamous cell carcinoma (HNSCC) cells and inhibited their invasiveness (22). Our study showed that treatment with A-type PAC DP-9 decreased expression and activation and induced nuclear translocation of EGFR in SKOV-3 cells dose-dependently. Association of EGFR with DNA-PK, which is involved in DNA repair, has been previously reported (23,24), and its nuclear translocation has been confirmed to modulate DNA repair caused by cisplatin or radiation in mouse fibroblast cell lines (25). Although EGFR nuclear translocation can be expected to activate DNA-PK as a counter measure to DNA damage due to quercetin and PAC-9 treatment, immunofluorescence microscopy analysis revealed downregulation of DNA-PK in ovarian cancer cells exposed to quercetin aglycone or PAC DP-9. The DNA-PK-mediated DNA repair that is induced by exposure to cisplatin in cancer cells is believed to be an important factor in reducing the efficacy of platinum-based chemotherapy (26,27). Therefore, the inhibition of DNA-PK expression in quercetin aglycone or PAC DP-9-treated ovarian cancer cells may partially account for the increased efficacy of cisplatin in ovarian cancer cells following quercetin aglycone or PAC DP-9 pre-treatment.

EGFR also regulates the extracellular signal-regulated kinase ERK-MAPK pathway to maintain normal cell growth, proliferation and differentiation (28). Aberrant activation of the EGFR-Ras-Raf-MEK-ERK cascade is believed to contribute to cancer development and progression (29). Both quercetin aglycone and PAC DP-9 downregulated activated Raf, ERK1/2 and MEK in OVCAR-8 cells, suggesting the ERK-MAPK pathway may be one of the anti-proliferative mechanisms. The Raf-MEK-ERK pathway is also known to control cell cycle progression through induction of key cell cycle regulatory factors such as cyclins, cyclin-dependent kinases (CDKs), and p21 (30). Phosphorylation of histone H3, which is believed to play an important role in cell division and oncogene induction, was shown to be stimulated by Ras-MAPK signaling pathway (31,32). In our study, we report different levels of upregulation of CDK inhibitors p18, p27, and most significantly, p21 after treatment with quercetin aglycone or PAC DP-9, as well as downregulation of cyclin D1 and phospho-histone H3, indicating their effects on cell cycle regulation. Expression of p21 can be induced by either p53-dependent or independent pathways (33,34). In SKOV-3 cells, where the p53 gene is mutated and loses its expression, PAC DP-9 induced p21 upregulation inversely correlated with the expression of ERK1/2, suggesting that PAC DP-9 modulated a p53-independent ERK pathway that mediates p21 regulation similar to a published mechanism in rhabdosarcoma cells (34).

Upregulation of CDK inhibitors and downregulation of cyclin D1 and phospho-histone H3 induced by the two cranberry flavonoids reflected the ability of quercetin aglycone and PAC DP-9 to cause cell cycle arrest in SKOV-3 and OVCAR-8 cells. At concentrations lower than IC_50_, both quercetin aglycone and PAC DP-9 induced G1/S phase cell cycle arrest in OVCAR-8 cells, consistent with the upregulation of cellular p21 that has been shown to inhibit CDK regulation in G1/S phase progression (35). On the other hand, SKOV-3 cells showed cell cycle arrest at G2/M phase within 24 h of treatment with quercetin aglycone or PAC DP-9, similar as shown previously (10). Interestingly, CDK2, which is inhibited by p21 and facilitates G1/S phase transition (35), was upregulated after treatment with quercetin aglycone or PAC DP-9 in both SKOV-3 and OVCAR-8 cells. We confirmed direct protein-protein interaction between EGFR and CDK2 in SKOV-3 cells through Co-IP, suggesting that CDK-2 overexpression could be induced by nuclearly localized EGFR to facilitate DNA repair and synthesis after exposure to quercetin aglycone or PAC DP-9. These observations suggest that cell division of the two ovarian cancer cell lines was regulated through different mechanisms by cranberry flavonoids. Targeting cell cycle checkpoints has been proposed as a promising approach to cancer treatment (36).

In conclusion, our study suggests that certain cranberry flavonols and PACs possess cytotoxic properties against ovarian cancer cells. The integration of quercetin and/or PAC DP-9 in ovarian cancer chemotherapy may provide for improved outcomes. Quercetin aglycone and PAC DP-9 induced cellular apoptotic events including cell cycle arrest and suppression of DNA repair pathways that highlight their potential as dietarily available therapeutic agents.

## Figures and Tables

**Table I:** Correlation coefficient (r), p-value and IC_50_ value of individual cranberry PACs and flavonols against SKOV-3 and OVCAR-8 cells.^a^

	SKOV-3			OVCAR-8		
		
Compound	Correlation coefficient	p-value	IC_50_ (μg/ml)	Correlation coefficient	p-value	IC_50_ (μg/ml)
DP-2	−0.917	0.0000263	194.8	−0.919	0.0000239	143.1
DP-3	−0.804	0.00163	210.8	−0.513	0.0882	ND
DP-4	−0.923	0.0000185	200.0	−0.936	0.00000741	164.1
DP-5	−0.983	1.15×10^−8^	126.3	−0.738	0.00616	209.9
DP-6	−0.929	0.0000129	201.1	−0.9	0.0000674	152.6
DP-7	−0.821	0.00106	288.7	−0.736	0.00631	211.2
DP-8	−0.944	0.00000397	137.9	−0.945	0.00000356	125.7
DP-9	−0.939	0.00000584	81.9	−0.923	0.0000189	137.8
DP-10	−0.494	0.103	ND	−0.565	0.0558	ND
DP-11	−0.966	3.53×10^−7^	165.9	−0.931	0.0000107	146.9
DP-12	−0.824	0.000978	162.6	−0.791	0.00216	248.5
M-Gal	0.331	0.293	ND	−0.78	0.00275	130.7
Q-Gal	−0.833	0.000757	164.8	−0.896	0.000082	132.7
Q-Glu	−0.899	0.0000684	160.6	−0.929	0.000013	160.6
Q-A-P	−0.665	0.0182	410.2	−0.777	0.00293	212.7
Q-A-F	−0.791	0.00217	173.5	−0.908	0.0000435	171.2
Q-Xylo	0.095	0.77	ND	−0.266	0.404	ND
Q-Rhamno	−0.879	0.000167	207.4	−0.945	0.00000369	163.4
Q-Aglycone	−0.982	1.45×10^−8^	83.2	−0.966	3.48×10^−7^	61.1

a Linear regression was conducted for each cranberry flavonoid between cell viability and compound concentration (untransformed and logarithm-transformed). IC_50_ value was calculated based on best-fit regression model (r closer to −1).

ND, not determined; M-Gal, myricetin-3-galactoside; Q-Gal, quercetin-3-galactoside; Q-Glu, quercetin-3-glucoside; Q-A-P, quercetin-3-arabinopyranoside; Q-A-F, quercetin-3-arabinofuranoside; Q-Xylo, quercetin-3-xylopyranoside; Q-Rhamno, quercetin-3-rhamnopyranoside; Q-Aglycone, quercetin aglycone.
